# Clarifying the biological and statistical assumptions of cross-sectional biological age predictors: an elaborate illustration using synthetic and real data

**DOI:** 10.1186/s12874-024-02181-x

**Published:** 2024-03-08

**Authors:** Marije H. Sluiskes, Jelle J. Goeman, Marian Beekman, P. Eline Slagboom, Hein Putter, Mar Rodríguez-Girondo

**Affiliations:** 1 Medical Statistics, Department of Biomedical Data Sciences, Leiden University Medical Center, Leiden, the Netherlands; 2 Molecular Epidemiology, Department of Biomedical Data Sciences, Leiden University Medical Center, Leiden, the Netherlands; 3 Max Planck Institute for the Biology of Ageing, Cologne, Germany

**Keywords:** Aging, Biological age, Aging divergence, Aging rate, Aging clocks, Cross-sectional biological age predictors, Klemera-Doubal

## Abstract

**Background:**

There is divergence in the rate at which people age. The concept of biological age is postulated to capture this variability, and hence to better represent an individual’s true global physiological state than chronological age. Biological age predictors are often generated based on cross-sectional data, using biochemical or molecular markers as predictor variables. It is assumed that the difference between chronological and predicted biological age is informative of one’s chronological age-independent aging divergence ∆.

**Methods:**

We investigated the statistical assumptions underlying the most popular cross-sectional biological age predictors, based on multiple linear regression, the Klemera-Doubal method or principal component analysis. We used synthetic and real data to illustrate the consequences if this assumption does not hold.

**Results:**

The most popular cross-sectional biological age predictors all use the same strong underlying assumption, namely that a candidate marker of aging’s association with chronological age is directly informative of its association with the aging rate ∆. We called this the identical-association assumption and proved that it is untestable in a cross-sectional setting. If this assumption does not hold, weights assigned to candidate markers of aging are uninformative, and no more signal may be captured than if markers would have been assigned weights at random.

**Conclusions:**

Cross-sectional methods for predicting biological age commonly use the untestable identical-association assumption, which previous literature in the field had never explicitly acknowledged. These methods have inherent limitations and may provide uninformative results, highlighting the importance of researchers exercising caution in the development and interpretation of cross-sectional biological age predictors.

**Supplementary Information:**

The online version contains supplementary material available at 10.1186/s12874-024-02181-x.

## Background

Individuals of the same chronological age show considerable variation in the rate at which they age: while some enjoy long and healthy lives, others experience early-onset functional decline, suffer from a range of diseases and die young [1]. This variability gave rise to the idea that, in addition to a chronological age, individuals also possess a biological age [2, 3]. This biological age should be an accurate reflection of one’s position on their life-course: when biological age exceeds chronological age this is indicative of accelerated aging (marking a higher physiological vulnerability, lower lifespan expectancy and increased risk to develop (multi)morbidity), the reverse of slow aging.

The question why, how and how fast we age is not only of biological interest, but has direct societal relevance. The enormous increase in average human lifespan that has been observed throughout most of the world in the last centuries has not been matched by an equal increase in healthspan (life years spent in good health) [1, 4]. This has led to a global healthcare burden, which is expected to only increase in the decades to come [5]. Measuring biological age could contribute to identifying individuals most at risk and helping them with targeted interventions. In addition, a better insight in the processes that underlie aging might help in designing interventions to slow down, delay or even reverse aging.

Biological age is latent: it cannot be directly measured, which complicates a direct evaluation of predictions. Many different operationalizations of this latent (and potentially multifaceted [6, 7]) concept are possible, and often not made explicit [8]. However, there is consensus that biological age, regardless of how it is exactly defined, should be a holistic measure of aging that contains information on aging above and beyond chronological age [9, 10]. We refer to the chronological age-independent part of biological age as the ‘aging divergence’ and denote it by the symbol ∆. Hence, we capture by aging divergence ∆ the difference between biological and chronological age (more precisely, biological age conditional on chronological age). Note that this quantity has also been referred to as the ‘aging rate’ or as the ‘age acceleration’, but these terms are less appropriate in a cross-sectional context, which is why we opt for the more neutral term ‘divergence’. We choose not to formally define biological age, as the key message of this paper holds for any definition of biological age that is based on the premise that the aging divergence ∆ contains information on one’s aging status above and beyond chronological age and that it is possible to predict (an aspect of) ∆.

In line with this consensus, predictions of biological age are generally evaluated by checking if the chronological age-independent part of a prediction, denoted by $\widehat{\Delta }$, is associated with time-to-death or other outcomes that are known to be measurable physiological outcomes representing the aging process (e.g., grip strength, frailty or cognitive function), in a model adjusted for chronological age.

The aging field is trying to detect (bio)markers indicative of the biological age of individuals, in this paper referred to as ‘candidate markers’ (of biological aging). Such candidate markers of biological aging must be informative of biological age beyond chronological age, i.e., they must be associated with one’s aging divergence ∆. Candidate markers can consist of molecular, biochemical, clinical or physiological health data. The earliest attempts to capture biological age made use of a limited number of physiological and biochemical markers [3, 11, 12]. More recently, the advent of high throughput bio-molecular technologies has resulted in the development of numerous high-dimensional omics-based age predictors. This renewed interest was initiated by the publication of the Horvath and Hannum DNA methylation (DNAm) age predictors [13, 14]. It was soon found that DNAm age predictions are associated with aging above and beyond chronological age [15–17]. Since then, various other omics-based age predictors have been developed, e.g., based on IgG glycomics [18], metabolomics [19], proteomics [20] or transcriptomics [21].

Biological age prediction methods, often referred to as ‘aging clocks’, can be divided in several generations. The first-generation prediction methods are based on the association of candidate markers of biological aging with chronological age. These methods hence require cross-sectional data only, where chronological age and candidate markers are measured at a single point in time. The second-generation prediction methods are based on the association of candidate markers with time-to-age-related-event data (as of yet, only time-to-mortality has been considered as outcome of interest). The three most well-known second-generation predictors are PhenoAge [22] and GrimAge [23], which both use DNAm marker data as (surrogate) predictor variables, and a mortality predictor named MetaboHealth [24], using metabolome data as predictor variables. It is speculated that the third generation will consist of prediction methods that are constructed using repeated measurement data, e.g., multiple measurements of the candidate predictors of interest [25]. Early examples of such methods are DunedinPoAm [26], which aims to measure the pace of biological aging, and DunedinPACE [27], which is constructed by regressing DunedinPoAm on DNAm marker data.

Although second- and third-generation epigenetic and metabolomics-based methods outperform first-generation (cross-sectional) methods in terms of their strength of association with time-to-mortality and other aging-related outcomes [25, 27–30], cross-sectional methods are still frequently developed, used and debated [31]. From a practical point of view, the ongoing popularity of cross-sectional methods can easily be explained: cross-sectional data are simply much more abundant than longitudinal (time-to-event) data. Moreover, the predicted aging divergences ∆ of several recent cross-sectional age predictors were found to be associated with time-to-mortality and the onset of other aging-related outcomes [15, 16, 19, 32].

The general consensus in the field therefore seems to be that even though cross-sectional biological age predictors are suboptimal, they still capture some signal related to biological aging, and can therefore still be of value. Nevertheless, how and under which assumptions they can capture this signal is not clear, neither from a statistical nor from a biological point of view. We believe that the statistical assumptions underlying these cross-sectional methods, and the consequences if they are not met, must be known and well understood for aging researchers to evaluate whether it makes sense use to such an approach. Lack of understanding of the assumptions and limitations of any prediction method can hamper progress in the field of biological age prediction and in the identification of relevant markers of aging. Though certain aspects of various cross-sectional methods have been sporadically criticized before (discussed in more detail in the next section), to the best of our knowledge an in-depth discussion of the key assumption that all cross-sectional approaches—often implicitly—use does not yet exist.

With this paper we attempt to fill that gap by considering this matter from several angles. We start by providing a comprehensive overview of the most popular cross-sectional biological age prediction methods. We discuss the assumption under which they are expected to work, namely that any marker’s association with chronological age is directly informative of its association with the age-independent part of the difference between predicted and chronological age, denoted by ∆. We call this the identical-association assumption and provide a theoretical result why this assumption is untestable. To illustrate the consequences in settings where this assumption does not (fully) hold, we use two synthetic data examples. Finally, we use real data to illustrate that caution must be taken when using cross-sectional data to predict biological age. With this we hope to increase awareness that all cross-sectional methods that either directly or indirectly use candidate markers’ correlation with chronological age may be superfluous, and in any case should not be used without carefully reflecting beforehand on the assumptions these methods make.

## Methods

### Overview of cross-sectional statistical approaches

By far the most popular statistical approach to estimate biological age (*B*) is to perform multiple linear regression (MLR) on cross-sectional data: chronological age (*C*) is taken as the outcome variable and regressed on a set of candidate markers of biological aging (*X*) that were measured at the same time as chronological age. Then the model’s predicted chronological age is considered to be informative of one’s biological age: $\widehat{B}=\widehat{C}= {\beta }_{0}+ \sum_{i=1}^{m}{\beta }_{i}{x}_{i}$, where *m* represents the number of candidate markers included in the regression and *x* represents a single marker. In this method predictions for the aging divergence ∆ are generally defined as the resulting residuals after regressing predicted biological age (i.e., $\widehat{C}$) on chronological age. Hence, the residuals of the chronological age model are considered to be informative of ∆. This approach is used with both low- and high-dimensional markers.

The MLR approach does not follow from an underlying model of biological age; however, it relies on a model that predicts chronological age to be indicative of the aging divergence ∆. For this to work it must hold that markers that are correlated with chronological age are also correlated with ∆, and vice versa. In fact, it is implicitly assumed that the higher the correlation with chronological age (in a multivariable model, so adjusting for all other included markers), the stronger it is correlated with ∆. Markers that are insignificant predictors of chronological age are assumed to be insignificant predictors of ∆.

Although the MLR approach is the most often-used cross-sectional approach, it has been criticized for various reasons. It suffers from inherent methodological problems, such as regression to the mean (fitted values regress towards the sample’s mean age such that biological ages calculated for those younger than the sample mean age tend to be too high and for those older, too low) and the so-called ‘biomarker paradox’ (a (bio)marker that perfectly correlates with chronological age is useless in estimating biological age) [33, 34]. The biomarker paradox is more than a mere theoretical danger: with epigenetic biological age predictors, in principle a nearly perfect chronological age predictor can be developed, as long as the sample size is large enough [35]. In such a case all signal related to biological aging would be lost. This paradox therefore illustrates the peculiarities that arise when the residuals of a linear regression are interpreted as meaningful quantities in their own right, while in the model formulation those residuals are per definition nothing but noise.

Alternative cross-sectional approaches have been proposed in an attempt to overcome some of these methodological issues. The most notable alternatives are principal component (PC)-based methods and the Klemera-Doubal (KD) method [36]. PC-based methods transform candidate markers to a set of uncorrelated principal components [37–40]. Most of the times, first a pre-selection of candidate markers is made based on how strongly each individual marker is correlated with chronological age. Often, the first principal component of this subset of variables is found to be correlated with chronological age and is hence interpreted as an ‘unscaled’ or ‘standardized’ biological age score *BS*. This score is sometimes transformed to an age-scale based on the mean and standard deviation of chronological age (*µ*_*C*_ and *σ*_*C*_) in the training sample: *B* = *BS* ∗ *σ*_*C*_ + *µ*_*C*_.

The Klemera-Doubal method [36] uses a reversed regression approach (regressing each candidate marker on chronological age). In contrast to the above methods, the KD method is based on an explicit underlying model of biological age. It assumes that the relation between biological age and chronological age can be expressed by *B* = *C* + ∆. Each marker *x* is governed by *B* but is also affected by random fluctuations. Assuming a linear relation between marker *x* and biological age, *x* equals *β*_0_ + *β*_1_ ∗ *B* + *ϵ*. This can also be expressed as *x* = *β*_0_ + *β*_1_ ∗ (*C* + ∆) + *ϵ*. That the coefficient *β*_1_ is the same for *C* and ∆ is a key assumption of the Klemera-Doubal method: in their model, a marker’s strength of association with chronological age is directly informative of its association with ∆. A biological age prediction is obtained by taking a linear combination of all included markers, each of them weighted in terms of the estimated slopes and residual variances resulting from the reversed regressions.

Though in certain settings the Klemera-Doubal method has been found to outperform MLR- and PC-based methods [41], extending the method to high-dimensional settings is not straightforward, since it assumes that all included markers are functionally uncorrelated. Therefore the KD method is primarily used in low-dimensional settings [38, 42, 43], or prior to applying the KD method principal component analysis is used to obtain a set of lower-dimensional markers [41, 44]. The limitations of the alternative cross-sectional approaches might explain the continued popularity of the MLR approach in high-dimensional settings. In a recent review of omics-based biological age predictors the Klemera-Doubal method is not mentioned and PC-based methods play a minor role [31].

### Reflection on the assumption underpinning cross-sectional biological age predictors

Use of the cross-sectional methods described above can be justified if a common assumption holds, namely that a candidate marker’s strength of association with chronological age is identical to its strength of association with one’s aging divergence (the chronological-age independent part of biological age) ∆. So by using one of the above cross-sectional methods for biological age prediction it is assumed that the traits most strongly associated with chronological age are the ones most informative of ∆. If a marker changes with chronological age irrespective of relevant changes in ∆, or vice versa, the assumption is not met. Note that this requires biological age to be defined as something other than simply the predicted chronological age: if not, a marker per definition cannot change with chronological age without changes in ∆. Then any trait associated with chronological age (e.g., percentage of grey hair) would per definition be a valid measure of biological age, which we believe to be a false premise.

For ease of reference, we henceforth refer to this shared assumption that the traits most strongly associated with chronological age are the ones most informative of ∆ as the *identical-association assumption*. The KD method explicitly makes this assumption. For the MLR approach the story is slightly more nuanced: as mentioned previously, this approach is not based on any underlying model of biological age, and it hence does not *explicitly* rely on any assumptions regarding biological age. One is therefore forced to ‘reverse engineer’ assumptions under which it is justified to use the MLR approach. One assumption under which this approach can be expected to work (i.e., captures signal related to ∆) is if the identical-association assumption holds. Markers with high absolute coefficient values will have a strong effect on the resulting chronological age prediction $\widehat{C}$, which is considered equal to biological age prediction $\widehat{B}$. The identical-association assumption is hence a sufficient assumption for the MLR approach, where it is a necessary one for the KD method. For the PC-based approaches this assumption is used when making a preselection of markers prior to finding the principal components, since only variables significantly correlated with chronological age are selected. It is therefore not surprising that the first principal component is often found to be correlated with chronological age: the variables were selected to share this common source of variance.

**Fig. 1:**
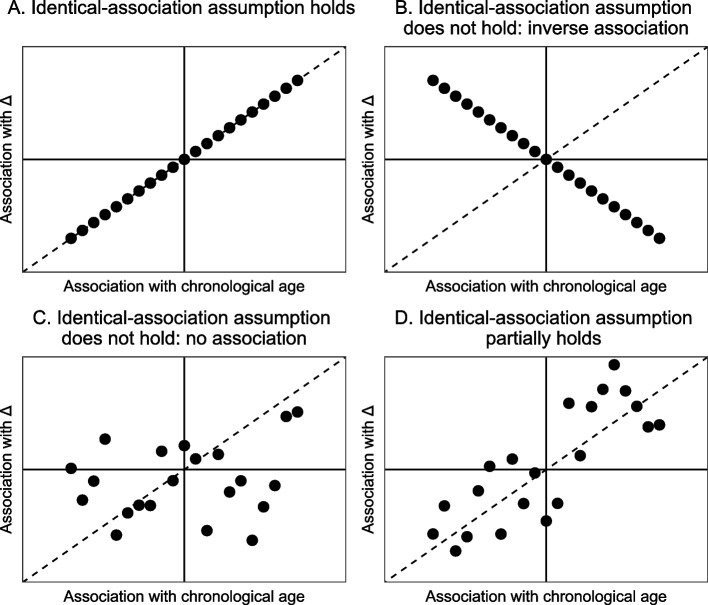
Conceptual visualization of the four scenarios. A scenario in which the identical-association assumption holds (**A**), a scenario in which the inverse relation holds (**B**), a scenario in which there is no association (**C**) and a scenario in which the identical-association assumption partially holds (**D**)

There are different degrees to which the identical-association assumption might hold in real data. For any set of candidate markers of biological aging, one can roughly distinguish four possible scenarios. The first scenario is that the identical-association assumption holds. If one would then plot the true association of markers with chronological age against their true association with aging divergence ∆, one would end up with a plot as given in the top left panel (A) of Fig. 1. (There are of course several ways to define ‘association’ – since we do not want to assume a specific model, we deliberately keep this term vague. The plots are therefore conceptual representations of the four scenarios.) As mentioned, the Klemera-Doubal method explicitly makes this assumption, as it assumes an identical regression coefficient (effect size) for chronological age and aging divergence ∆ and no other sources of shared variance. In this first scenario it would make perfect sense to use a cross-sectional prediction method. The second scenario (shown in panel B of Fig. 1) is one in which the opposite of the identical-association assumption holds: the stronger a marker is positively associated with chronological age, the stronger it is negatively associated with aging divergence ∆. This is an unlikely possibility, which is only included such that the four scenarios discussed here are collectively exhaustive. The third scenario (shown in panel C of Fig. 1) is that the markers’ strength of association with chronological age is not informative of their association with aging divergence ∆ at all. In such a scenario, using a cross-sectional prediction method would be useless: the weights that cross-sectional methods give to markers will be based on their association (both strength and direction) with chronological age, but these weights will be completely uninformative of the markers’ association with ∆. The fourth and final possibility (shown in panel D of Fig. 1) is that the markers’ strength of association with chronological age is somewhat, but not exactly, informative of their association with aging divergence ∆. Of the four scenarios this appears to be the most realistic one.

**Fig. 2:**
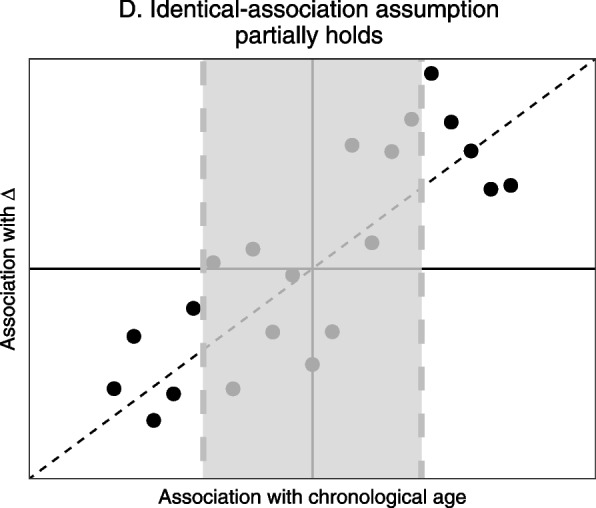
Zoomed-in version of the bottom right panel of Fig. 1. If markers are (pre-)selected based on their strength of correlation with chronological age, those in the grey area (i.e., those most weakly associated with chronological age) are not selected

For this fourth scenario it is important to remember that many of the high-dimensional cross-sectional biological age predictors perform some kind of marker selection, either before including them in the model or during the model fitting itself. If one would then only include the variables most strongly correlated with chronological age (i.e., only the edges of Fig. 1D would be included, as illustrated in Fig. 2), there no longer is a relation between strength of association with chronological age and with aging divergence ∆. However, in Fig. 2 there still is a relation between the *direction* of the association of the selected markers with chronological age and with ∆. This suggests that in a scenario where the fourth scenario holds and candidate markers of biological aging have been pre-selected, the size of a candidate marker’s association with chronological age will not be informative of its association with ∆, but the sign (positive/negative) of this association will be.

**Fig. 3:**
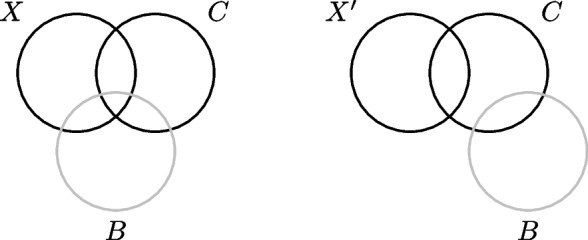
Venn diagrams illustrating the variance shared between biological age (B), chronological age (C) and the candidate markers of biological aging X (true, left diagram) and X’ (false, right diagram). Black indicates observed variance; grey unobserved

Which scenario holds in a given data set determines whether or not it makes sense to use a cross-sectional method to predict biological age. Unfortunately, in cross-sectional data the identical-association assumption cannot be proven or disproven, because it is untestable: it is impossible to tell to what extent a marker is associated with aging divergence ∆ based on its association with chronological age alone. For a formal theorem and proof of the untestability of the identical-association assumption we refer to (Additional file 1). An intuitive visualization of the proof is given in Fig. 3. It shows correlation Venn diagrams [45] for two candidate markers of biological age, *X* and *X*′. The two candidate markers have the same association with chronological age *C*, but where marker *X* shares association with biological age *B*, candidate marker *X*′ has no such association. Since *B* is unobserved, we only have information on the joint distribution of *X* and *C*, or *X*′ and *C*, respectively. With respect to this observable variation, the diagrams for *X* and *X*′ are identical. It follows that we cannot distinguish between the true marker *X* of biological age and the false marker *X*′. Hence, if the identical-association assumption does not hold, it is impossible to distinguish true markers of ∆ from false ones. Using cross-sectional biological age prediction methods, thereby (implicitly) believing in the identical-association assumption, is therefore based on biological hope or knowledge alone, not on a statistical property of the cross-sectional methods.

Whether or not it is justified to believe in the identical-association assumption will depend on the situation at hand. In the real data illustration section of this paper we provide an example where this assumption turns out not to hold. The discussion section contains several other examples of scenarios where this assumption is likely not to hold.

## Results

### Two illustrative examples

This section contains two synthetic data examples that illustrate two aspects of the identical-association assumption.

#### Example 1: untestability of the identical-association assumption

We created a synthetic data set with four variables: chronological age *C*, biological age *B*, true marker of biological age *X* and false marker of biological age *X*′. *X* and *X*′ follow the same distribution and have the same strength of correlation with *C*. We based our data generation approach on the type of additive model proposed by Klemera and Doubal [36]. We generated *n* observations as follows. Independently generate the following elements:

- *C* ~ *N(µ, *$${\sigma }_{c}^{2}$$*);*
- *∆* ~ *N(0, *$${\sigma }_{\Delta }^{2}$$*);*
- *Λ* ~ *N(0, *$${\sigma }_{\Lambda }^{2}$$*);*
- *ϵ* ~ *N(0, *$${\sigma }^{2}$$*);*
- *ϵ′* ~ *N(0, *$${\sigma }^{2}$$*).*

From these elements, construct:

- *B* = *C* + *∆;*
- *X* = *α* + *β* × *(C* + *∆)* + *ϵ (*= *α* + *β* × *B* + *ϵ);*
- *X′* = *α* + *β* × *(C* + *Λ)* + *ϵ′.*

**Fig. 4:**
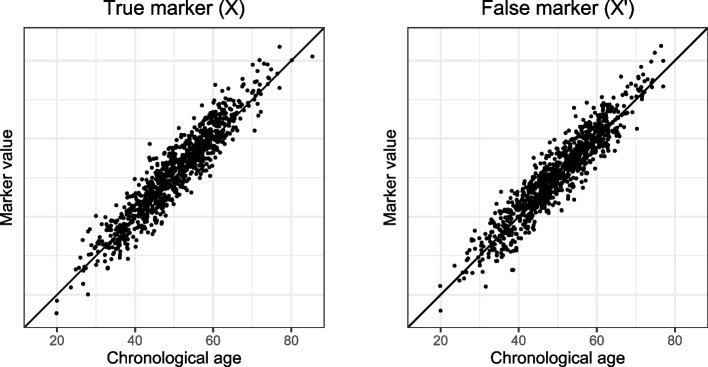
Chronological age plotted against the marker value for true marker X and false marker X′

**Fig. 5:**
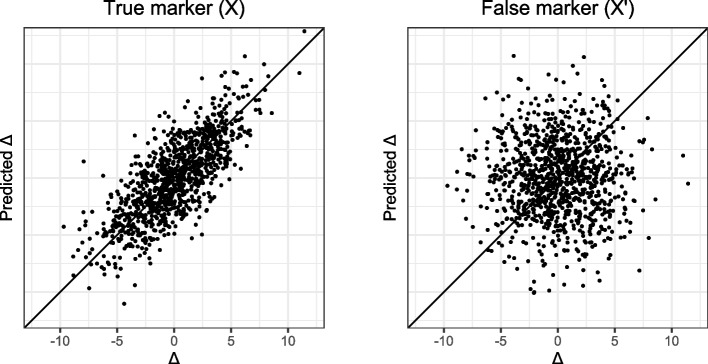
Aging divergence Δ plotted against predicted Δ for true marker X and false marker X′. The biological age predictions were obtained using linear regression

We used the following parameter values: *n* = 1000*, μ* = 50*,*${\sigma }_{c}^{2}$= 10*,*${\sigma }^{2}$= 2*,*${\sigma }_{\Delta }^{2}$ = ${\sigma }_{\Lambda }^{2}$  = 3*, α* = 1*, β* = 1. *X* and *X*′ have the same distribution and the same relation with chronological age, as seen in Fig. 4. However, *X* correlates with the individual aging divergence ∆ while *X*′ does not, as seen in Fig. 5. This implies that *X* has useful information on biological age that is not already in chronological age while *X*′ does not. However, in real cross-sectional data ∆ is not observed: with respect to their association with the observable variable chronological age these two candidate markers are identical, as can be seen in Fig. 4.

Since the observable data (*X,C*) and (*X*′*,C*) are indistinguishable from each other, any method we would apply on either (*X,C*) or (*X*′*,C*) would assign the same weight to either *X* or *X*′. This holds for the linear regression method, as is clear from Fig. 5. It also holds for the Klemera-Doubal method, since that method would assign the same weights to both *X*′ and *X*. Principal components-based methods would not be able to distinguish an informative source of variance (i.e., ∆) from an uninformative source of variance (here denoted by Λ). In fact, no cross-sectional method can distinguish between *X* and *X*′ based on their association with chronological age *C*, because the identical-association assumption is untestable. Therefore, no cross-sectional method can provide evidence that a candidate marker is a truly informative *X* rather than a completely uninformative *X*′.

#### Example 2: consequences of believing in the identical-association assumption under the four different scenarios

The first example illustrated that cross-sectional methods cannot be relied upon to select true markers of the aging divergence ∆. Nevertheless, predicted ∆-values of several cross-sectional age predictors have been found to be associated with time-to-mortality and several other age-related outcomes [31], albeit often weakly. This can only be the case if a marker’s strength of correlation with chronological age is at least somewhat indicative of its strength of association with true aging divergence ∆.

To illustrate this, we generated a possible realization of each of the four conceptual scenarios depicted in Fig. 1. We obtained predictions for aging divergence ∆ using multiple linear regression (MLR) and the KD method. We know that if the identical-association assumption does not hold, the weights found by MLR and the KD method are uninformative of a marker’s strength of association with ∆. If the identical-association assumption partially holds, the size of the weights that cross-sectional methods assign to markers will not be informative but the signs (positive/negative direction) of these weights still are (Fig. 2). We illustrate this by also including a third, ‘naive’ prediction method in this second example, where similar to the MLR approach we took a linear combination of markers. In this third prediction method each marker was assigned the same weight, namely the mean of the MLR coefficients. The sign of each coefficient was kept unchanged, because we generally expected the sign to be correct. We included this third approach to illustrate that if the identical-association assumption does not hold, weights obtained using the MLR or Klemera-Doubal method might result in less accurate predictions than naively assigning each marker the same weight.

For this second example we generated four data sets, *DF*_*A*_, *DF*_*B*_, *DF*_*C*_ and *DF*_*D*_, corresponding to the scenarios in Fig. 1. To keep it simple, each data set has only three markers(*X*_1_, *X*_2_ and *X*_3_), which are associated with chronological age *C* and with aging divergence ∆ to varying degrees in each of the four scenarios. We generated *n* observations as follows. Independently generate:

- *C* ∼ *N*(*µ,*$${\sigma }_{c}^{2}$$);
- *Δ* ~ *N(0, *$${\sigma }_{\Delta }^{2}$$*);*
- *ϵ*_*i*_ ∼ *N*(0*, *$${\sigma }_{i}^{2}$$*)*.

Construct biological age:

- *B* = *C* + ∆.

Construct markers:

- *X*_1_ = *β*_*C,*1_ × *C* + *β*_∆*,*1_ × ∆ + *ϵ*_1_;
- *X*_2_ = *β*_*C,*2_ × *C* + *β*_∆*,*2_ × ∆ + *ϵ*_2_;
- *X*_3_ = *β*_*C,*3_ × *C* + *β*_∆*,*3_ × ∆ + *ϵ*_3_.

Per scenario, the values chosen for *β*_*C,*1_ and *β*_∆*,*1_ can be found in Table 1. The following parameter values were used in all four scenarios: *n* = 1000*, µ* = 50*,*${\sigma }_{c}^{2}$ = 10 and ${\sigma }_{\Delta }^{2}$ = 5. The standard deviation of the errors *ϵ* were chosen such that the relation between the (scaled and centered) three markers and chronological age is the same in in all four data sets (see Additional file 2). Hence, based on the observable variables alone (*X*_1_, *X*_2_, *X*_3_ and *C*) the four data sets are indistinguishable.

**Table 1:** The coefficients used to construct the markers X_1_, X_2_ and X_3_ for the four different scenarios (A-D) as presented in synthetic data example 2

	**X**_**1**_		**X**_**2**_		**X**_**3**_	
	${\beta }_{c}$	${\beta }_{\Delta }$	${\beta }_{c}$	${\beta }_{\Delta }$	${\beta }_{c}$	${\beta }_{\Delta }$
**Scenario A**	10	10	3	3	5	5
**Scenario B**	10	-10	3	-3	5	-5
**Scenario C**	10	0	3	10	5	-1
**Scenario D**	10	9	3	5	5	5

**Fig. 6:**
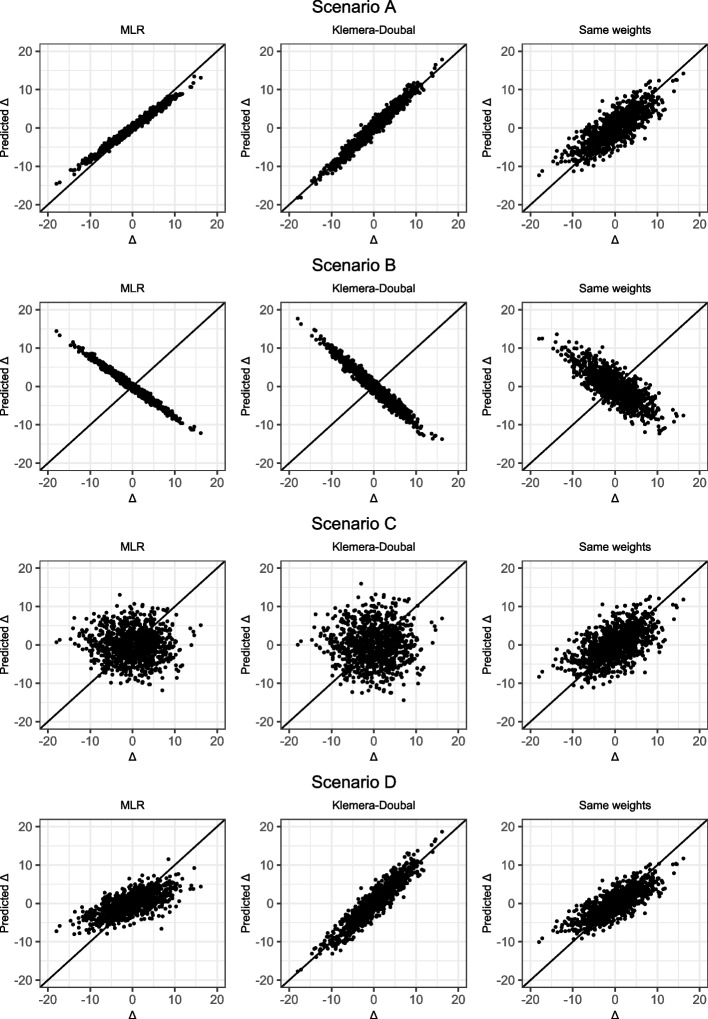
Aging divergence Δ plotted against predicted Δ in synthetic data example 2 for all four scenarios (A, B, C and D). Results are presented for the MLR approach, the Klemera-Doubal method and the MLR approach where each marker is assigned the same weight

If the identical-association assumption holds (scenario A), the MLR approach and the Klemera-Doubal approach outperform the equal weights approach (Fig. 6, first row: the closer the points are to the diagonal line ∆ = predicted ∆, the better the performance of the method). In this case a marker’s association with chronological age is directly informative of its association with aging divergence ∆, so any method that weighs markers according to their strength of correlation with chronological age will do well. In the unrealistic case that a marker’s association with chronological age is inversely related to its association with aging divergence ∆ (scenario B), all methods will perform badly, as is to be expected (Fig. 6, second row). If there is no relation between a marker’s association with chronological age and its association with aging divergence ∆ (scenario C), the equal weights approach outperforms the two cross-sectional approaches, which appear to capture only noise (Fig. 6, third row). In our realization of scenario D, it can be seen that all methods capture some signal (Fig. 6, fourth row). The Klemera-Doubal method does best, but that might not be surprising given the data generation approach, which was based on the type of additive model Klemera and Doubal assume. Interesting is that the same weights approach outperforms the MLR-approach. Naturally, this is just one possible realization: with different values for *β*_*C*_ and *β*_∆_ the coin could flip in favor of the MLR-method over the equal weights method (and with a different data generation mechanism, possibly also over the KD method).

### Real data illustration

The insights gained from the synthetic data scenarios are of immediate practical relevance. We illustrate this with a real data illustration.

We used data from the Leiden Longevity Study (LLS) [46]. The LLS follows long-lived siblings of Caucasian descent, their offspring and the partners of their offspring. We used data on the offspring and partners (*N* = 2312). Participants who were lost to follow-up (*N* = 10) or who had at least one missing metabolite value (*N* = 37) were excluded. In total 1593 offspring and 674 partners were included, of which 998 men and 1269 women (mean age at inclusion 59.2 years, sd 6.7). Participants were included between March 2002 and May 2006. Registry-based follow-up until November 2021 was available. Median follow-up time was 16.3 years (IQR: 15.3–17.1). 309 deaths were observed. The Medical Ethics Committee of the Leiden University Medical Center approved the study and informed consent was obtained from all participants.

As candidate markers of biological aging we used blood-based metabolic variables. The metabolic variables were quantified using a well-standardized high-throughput nuclear magnetic resonance (^1^H-NMR) metabolomics platform [47, 48] of Nightingale Health Ltd. (Helsinki, Finland). Of the more than 200 metabolic variables available, a subset of 59 was selected, previously found to be most reliable and independent [24] and used in various subsequent publications [19, 49]. Prior to analysis, a small constant was added to all metabolic variables after which they were log-transformed and scaled.

The complete two-generation Leiden Longevity Study has previously been used in two major analyses by our group, constructing biological age predictors (on cross-sectional as well as time-to-event basis) based on the same metabolic variables in much larger data sets. From these studies we observed that the constructed predictors as well as many of the 59 metabolic variables separately were predictive of prospective mortality [19, 24].

To illustrate the problems that can arise when using cross-sectional methods to predict biological age, we took a similar approach as in synthetic data example 2: we contrasted an often-used cross-sectional approach to obtain predictions for aging divergence ∆—in this case penalized regression, hereafter denoted by method 1—with naive methods to obtain predictions for ∆—in this case first selecting metabolites univariately associated with chronological age and then using (unpenalized) multiple linear regression (method 2), a linear combination with either equal weights (method 3) or randomly drawn weights (method 4).

For each of the four methods, predictions for aging divergence ∆ were obtained as follows. For method 1 we first obtained an age prediction using penalized MLR with a ridge penalty. Using 10-fold cross-validation, the penalization parameter *λ* was chosen such that the mean cross-validated error was minimized. Chronological age was taken as the outcome variable and all 59 metabolic variables were included as predictor variables. In method 2 we performed (unpenalized) multiple linear regression on a subset of variables correlated with chronological age. 26 of the 59 metabolic variables were significantly correlated with chronological age, using a Bonferroni-corrected significance threshold of 0*.*05*/*59 = 8*.*47 × 10^−4^. For method 3 we again took a linear combination of the 26 metabolic variables significantly correlated with chronological age. Here we assigned each variable same weight, namely the mean of the absolute value of the MLR-coefficients from method 2 (excluding the intercept). Although the coefficients were averaged, the *sign* of each variable’s coefficient was kept, for the same reason as illustrated by Fig. 2: in general we deem it unlikely that a variable is positively correlated with chronological age but negatively with ∆, though exceptions, for example due to compensatory processes, may exist. Method 4 is a variation on method 3: 1,000 different linear combinations of the same 26 variables were taken, where each variable was assigned a coefficient randomly drawn from a uniform distribution. Similar to method 3, the weights were drawn at random but the signs were kept. For each of the four methods, predictions for ∆ were obtained by regressing the linear combination of metabolic variables (the fitted values) on chronological age and obtaining the residuals.

We then compared the performance of the four methods by scaling the predictions for aging divergence ∆ obtained using each of the four methods and including them in a Cox proportional hazards (PH) model with time-to-mortality as outcome. This is a common approach to check the validity of ∆-predictions if data on time-to-death is available [15, 16, 19, 25, 28, 32, 50]. We used chronological age as the timescale of the Cox PH model, taking delayed entry into account by including age-at-baseline as the left truncation variable, and adjusted for sex. Since all predicted ∆-values were scaled prior to inclusion, the higher the coefficient for ∆, the stronger the association with time-to-mortality.

**Fig. 7:**
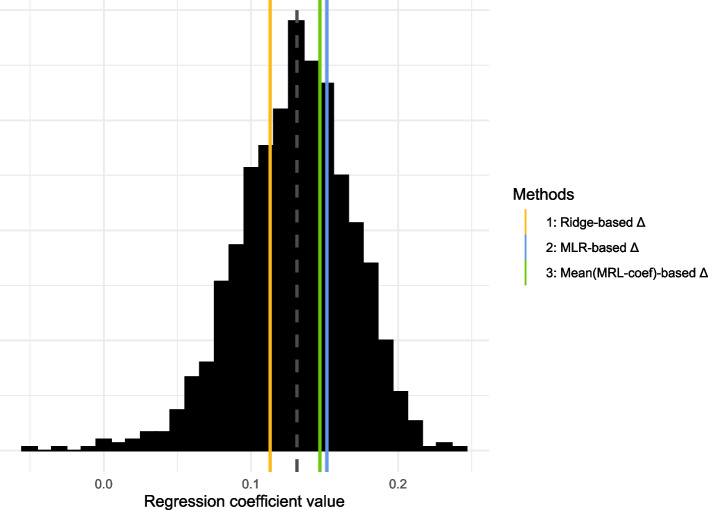
Regression coefficients (effect sizes) of the predicted Δ-values in a Cox PH model with time-to-mortality as the outcome, using the LLS data and 59 metabolic variables as predictor variables. The predicted Δ-values were calculated using 4 methods: using ridge regression (method 1), using multiple linear regression on a subset of metabolites (method 2), taking a linear combination where each metabolic variable was assigned the same weight (method 3), and taking a linear combination where each metabolic variable was assigned a weight randomly drawn from a standard uniform distribution, repeated 1,000 times (histogram, method 4; median of histogram values denoted by grey dashed line)

The Cox PH coefficients of the different ∆-predictions (i.e., the effect sizes of the association with prospective mortality) obtained with these four methods are compared in Fig. 7. It can be seen that the coefficient for aging divergence ∆ obtained with method 1 is lower than those of methods 2 and 3: hence, association with time-to-death is weaker. The blue and green lines of methods 2 and 3 are very close to each other: using multiple linear regression (method 2) works just as well as assigning each marker the same coefficient (method 3). The histogram represents the distribution of the 1,000 coefficients obtained by assigning each metabolic variable a randomly drawn weight (method 4), repeated 1,000 times. More than half of the histogram area is to the right of the yellow line of the ridge-based coefficient (method 1), and a substantial part is even to the right of the blue and green lines of methods 2 and 3. These results imply that in this particular setting, the naive methods capture more signal related to prospective mortality than the ‘proper’ cross-sectional method 1.

Although Fig. 7 shows that predictions for aging divergence ∆ obtained via ridge regression on 59 metabolic variables (method 1) are less strongly associated with mortality than predictions for ∆ obtained using standard multiple linear regression on 26 metabolic variables (method 2), the *chronological* age predictions obtained with method 1 are more accurate than the ones obtained with method 2 (root-mean-square error method 1: 6.01, root-mean-square error method 2: 6.18). This exemplifies the biomarker paradox: improved chronological age predictions do not imply improved biological age predictions. In fact, after a certain point the association will weaken. We see that the better chronological age prediction performance of method 1 already results in a weaker association of ∆ with prospective mortality.

Note that all coefficients in Fig. 7 are positive. Since we kept the coefficient signs of method 2 for methods 3 and 4, it confirms our earlier assertion that if a marker is positively associated with chronological age, it is unlikely to be negatively associated with aging divergence ∆ (and vice versa). This explains why despite the suboptimality of cross-sectional methods, cross-sectional ∆-predictions have repeatedly been found to be associated with prospective mortality and other age-related outcomes [15, 16, 19, 32]—albeit (much) weaker than second-generation biological age predictors [25, 28–30]. The direction of the coefficients contains information regarding the signal. However, one must realize that unless the identical-association assumption (almost fully) holds, no more signal will be captured with cross-sectional methods than if markers would have been assigned weights at random.

## Discussion

We have shown that the most popular cross-sectional biological age predictors, where candidate markers of biological aging and chronological age are measured at a single point in time, rely on the same underlying assumption to justify their use: a candidate marker’s strength of association with chronological age should be directly indicative of its strength of association with the difference between biological and chronological age, also known as one’s aging divergence ∆. We have called this assumption the identical-association assumption. We noted that there is no inherent statistical reason why a candidate marker’s association with chronological age *C* is indicative of its association with ∆: this depends on the biological context. Importantly, as we have proven, whether the identical-association assumption holds is untestable in a cross-sectional setting. As a consequence, one cannot distinguish true markers of biological age from false ones in such settings. A candidate marker can be correlated with chronological age but be completely uninformative of ∆. The opposite holds as well: a candidate marker may not be associated with chronological age, while being a true marker for ∆. We illustrated that unless chronological age and ∆ are equally strongly associated with each marker, there is no guarantee that the size of the weights that a cross-sectional method assigns to candidate markers is informative of the underlying truth. The identical-association assumption did not hold in the empirical data we considered. It should however be noted that we worked with a single real data set which is limited in size and scope. Our real data section is therefore primarily meant as an illustration of the potential practical consequences of constructing a cross-sectional biological age predictor if the identical-association assumption does not hold. It does not provide evidence for or against the extent to which this assumption holds in larger data sets or data sets with other types of candidate markers. Still, there is evidence that the identical-association assumption also does not hold in DNAm data: Levine et al. [22] regressed a phenotypic age measure that captured differences in lifespan and healthspan on CpG-sites and found that the CpG-sites with the highest resulting weights did not correlate with chronological age at all. One can think of other realistic scenarios in which the strength of association of candidate markers of aging diverges between chronological age and ∆: the strength of association of a true marker of biological age with either chronological age or ∆ might change in different periods of the lifespan, might be non-linear, could be subject to cohort effects or measurement error, et cetera.

In this paper we chose not to provide a formal definition or operationalization of biological age, as the key message of this paper holds for any definition of biological age that is based on the premise that there exists such a thing as (a possibly multi-dimensional) biological age, that the aging divergence ∆ contains information on one’s aging status above and beyond chronological age and that it is possible to predict (an aspect of) ∆. We are aware that aging is generally considered a multi-faceted process [6, 7] and that some researchers question whether there exists a single unitary biological age, as aging is likely not a single biological phenomenon [51]. This is an interesting debate in itself: whether or not one finds it reasonable to believe in the existence of (one or more) latent biological age(s) should ideally precede any methodological considerations, such as those discussed in this paper. But as cross-sectional methods are frequently used for the prediction of biological age, we believe there is a value in discussing their underlying assumptions in depth.

We are not the first to consider which (statistical) assumptions must hold in order for a cross-sectional biological age predictor to be truly informative: Hertel and al. [52] discussed with great mathematical rigor the slightly more general, but closely related problem which statistical constraints must hold for the prediction error of prediction scores (in this context: the prediction error of predicted chronological age) to be informative about hidden biological traits (in this context: biological age). Similar conclusions are drawn: Hertel and al. also stress that it is not a sensible procedure to maximize model fit (they use the illustrative term ‘conceptual overfitting’ for the previously mentioned biomarker paradox) and state that ideally, predictor selection should be done on theoretical grounds only. In that, the scope of their work differs from ours: we focus solely on biological age prediction and discuss the assumption that must hold if one starts with a large number of candidate predictors from which true markers of aging are to be identified (as is common within the biological aging field) instead of starting from a set of known true markers.

Recently Nelson et al. [53] addressed another important concern related to identification of aging markers based on cross-sectional data: mortality selection can bias the identification of markers, up to a point where cross-sectional analyses are less likely to identify true markers than if markers had been selected at random. While Nelson et al. [53] state that this issue can be circumvented by only including markers that are known to be truly associated with mortality, in our second synthetic data example we illustrated that even in cases where all candidate markers are truly associated with biological age given chronological age, cross-sectional methods might not contribute either to selecting markers or to proving their validity.

We would like to stress that we do not claim that cross-sectional predictors of biological age cannot capture any signal. Although the identical-association assumption might not be realistic, for some (perhaps most) candidate markers the direction of a marker’s association with chronological age can still be informative. This also explains why many cross-sectional clocks were indeed found to be (weakly) correlated with various age-related outcomes [15, 16, 19, 32]: the sign of a candidate marker’s association with chronological age can be informative or uninformative of its association with aging divergence ∆, but it is unlikely to be counter-informative. Hence, most cross-sectional methods can be expected to still capture some signal—but potentially not better than any other approach that in some naive or random way assigns weights to markers associated with chronological age. We do not reject the possibility that markers exist for which the identical-association assumption does hold. This assumption may or may not hold for different types of markers, but in a cross-sectional setting there is no way to tell.

Since there is no way in which the quality of a biological age predictor can be assessed using cross-sectional data alone, it follows that there is no way to optimize the quality of biological age predictions using cross-sectional data. Therefore, it is likely that biological age predictors based on cross-sectional data are highly suboptimal—they primarily capture signals related to chronological age, as also remarked by [31]—and that much better predictors could be constructed if researchers could work directly with longitudinal data.

This raises the question whether cross-sectional methods still have a place in the biological aging prediction landscape, or whether they should be abandoned completely in favor of methods that use longitudinal (time-to-mortality) data [22–24]. By making the reasonable assumption that a higher biological age corresponds to a higher mortality risk, these time-to-mortality-based methods overcome the testability issue inherent to cross-sectional methods. The track record of these prospective mortality-trained methods in predicting various aging-related outcomes is indeed better than that of cross-sectional ones [25, 28–30]. Nevertheless, due to the relative abundance of cross-sectional data over longitudinal (time-to-event) data, cross-sectional predictors of biological age remain popular [31]. We think cross-sectional data can still play a role if the number of candidate markers is too high for to the limited sample size of the longitudinal data that is available and/or if there is little prior knowledge on the association between the candidate markers under consideration and aging divergence ∆, which in this new era of high-dimensional omics-based aging clocks is quite a likely scenario. In such a case, cross-sectional data could be used to make a pre-selection of markers most strongly correlated with chronological age, as one might reasonably expect that at least part of these candidate markers will also be strongly correlated with ∆. Such a pre-selection does not have to be conducted in a multivariate way, but can be done per marker, as we did in our real data illustration.

Our view is that if longitudinal (aging-related outcome) data is available, methods using this information are to be preferred above cross-sectional ones to develop a biological age predictor. Depending on the extent to which the identical-association assumption holds in the data set under consideration, longitudinal methods might be preferred even if the sample size of the available longitudinal data is much smaller. Furthermore, we believe that the sizes of the coefficients of candidate markers obtained with cross-sectional methods should neither be used nor interpreted. If researchers do decide to develop a biological age predictor based on cross-sectional data only, they should be explicit about the underlying assumptions of the method they used and to what extent these assumptions are expected to hold.

## Conclusions

In conclusion, we discussed that the most popular cross-sectional biological age predictors all use the same underlying assumption, which we have called the identical-association assumption. This assumption is untestable in a cross-sectional setting. There is no statistical reason why this assumption should hold: it depends on the biological context. If it does not hold, weights assigned to candidate markers of aging are uninformative of the underlying truth, and no more signal may be captured than if markers would have been assigned weights at random. Any researcher interested in developing, using or interpreting cross-sectional models of biological age should be aware of the inherent limitations of these models.

### Supplementary Information

**Supplementary Material 1.**

**Supplementary Material 2.**

## Data Availability

All R-code used for the analyses in this paper, including the code used to generate the synthetic data, is available in a public GitHub repository (https://github.com/marije-sluiskes/cross-sectional-bioage). Access to the individual-level data from the Leiden Longevity Study is restricted based on privacy regulations and informed consent of the participants. These data hence cannot be made available in a public repository. Data of the Leiden Longevity Study may be made available to researchers upon reasonable request to Eline Slagboom (p.slagboom@lumc.nl) or Marian Beekman (m.beekman@lumc.nl).

## Abbreviations

B: Biological age
C: Chronological age
∆: Aging divergence
X: Candidate marker of biological age
PC: Principal components
MLR: Multiple linear regression
KD: Klemera-Doubal
LLS: Leiden Longevity Study
