# Supplementary material for: Clarifying the biological and statistical assumptions of cross-sectional biological age predictors: an elaborate illustration using synthetic and real data
Source: BMC Med Res Methodol. 2024 Mar 8;24:58. doi: 10.1186/s12874-024-02181-x (PMC10921716; doi:10.1186/s12874-024-02181-x)
Supplement: Supplementary file 1 — Supplementary Material 1. [file 12874_2024_2181_MOESM1_ESM.pdf]

SUPPLEMENTARY NOTE 1

This Supplementary Note contains the theoretical foundation underpinning our statement that in a cross-sectional setting the identical-association assumption is untestable.

Denote by C chronological age, by B biological age and by X a true marker of biological age given chronological age $(B|C)$.

Theorem. *For every triplet (X, C, B) of continuous random variables, there exists another continuous random variable X' such that $(X', C) \stackrel{d}{=} (X, C)$ and X' is independent of B given C.*

Proof. Denote by $f(x, c, b)$ the joint density of (X, C, B). Let

$$f'(x, c, b) = \frac{\int_{-\infty}^{\infty} f(x, c, b)dx \int_{-\infty}^{\infty} f(x, c, b)db}{\int_{-\infty}^{\infty} \int_{-\infty}^{\infty} f(x, c, b)dbdx}$$

be the joint density of X', C, B. As the integral of $f'(x, c, b)$ over the entire space equals 1, this also constitutes a proper joint density. Moreover, this density is consistent with f, since

$$\int_{-\infty}^{\infty} f'(x, c, b)dx = \int_{-\infty}^{\infty} f(x, c, b)dx.$$

We have that $(X', C) \stackrel{d}{=} (X, C)$, since

$$\int_{-\infty}^{\infty} f'(x, c, b)db = \int_{-\infty}^{\infty} f(x, c, b)db.$$

Further, we have that X' is independent of B given C since $f'(x, c, b) = g(b, c)h(x, c)$ where

$$g(b, c) = \frac{\int_{-\infty}^{\infty} f(x, c, b)dx}{\int_{-\infty}^{\infty} \int_{-\infty}^{\infty} f(x, c, b)dbdx},$$

and $h(x, c) = \int_{-\infty}^{\infty} f(x, c, b)db$. □

Here we have considered a scenario with only one marker (X). In practice, researchers have many candidate markers to choose from, which are typically combined to a single biological age-metric. In that case, X or X' can be viewed as the resulting biological age metrics. The theorem then asserts that we cannot distinguish between a good metric X and a bad metric X'.

We emphasize that the result of this theorem is independent of the method used to infer on biological age: such inference is impossible with any cross-sectional method.
