# Supplementary material for: Clarifying the biological and statistical assumptions of cross-sectional biological age predictors: an elaborate illustration using synthetic and real data
Source: BMC Med Res Methodol. 2024 Mar 8;24:58. doi: 10.1186/s12874-024-02181-x (PMC10921716; doi:10.1186/s12874-024-02181-x)
Supplement: Supplementary file 2 — Supplementary Material 2. [file 12874_2024_2181_MOESM2_ESM.pdf]

SUPPLEMENTARY FIGURE 1

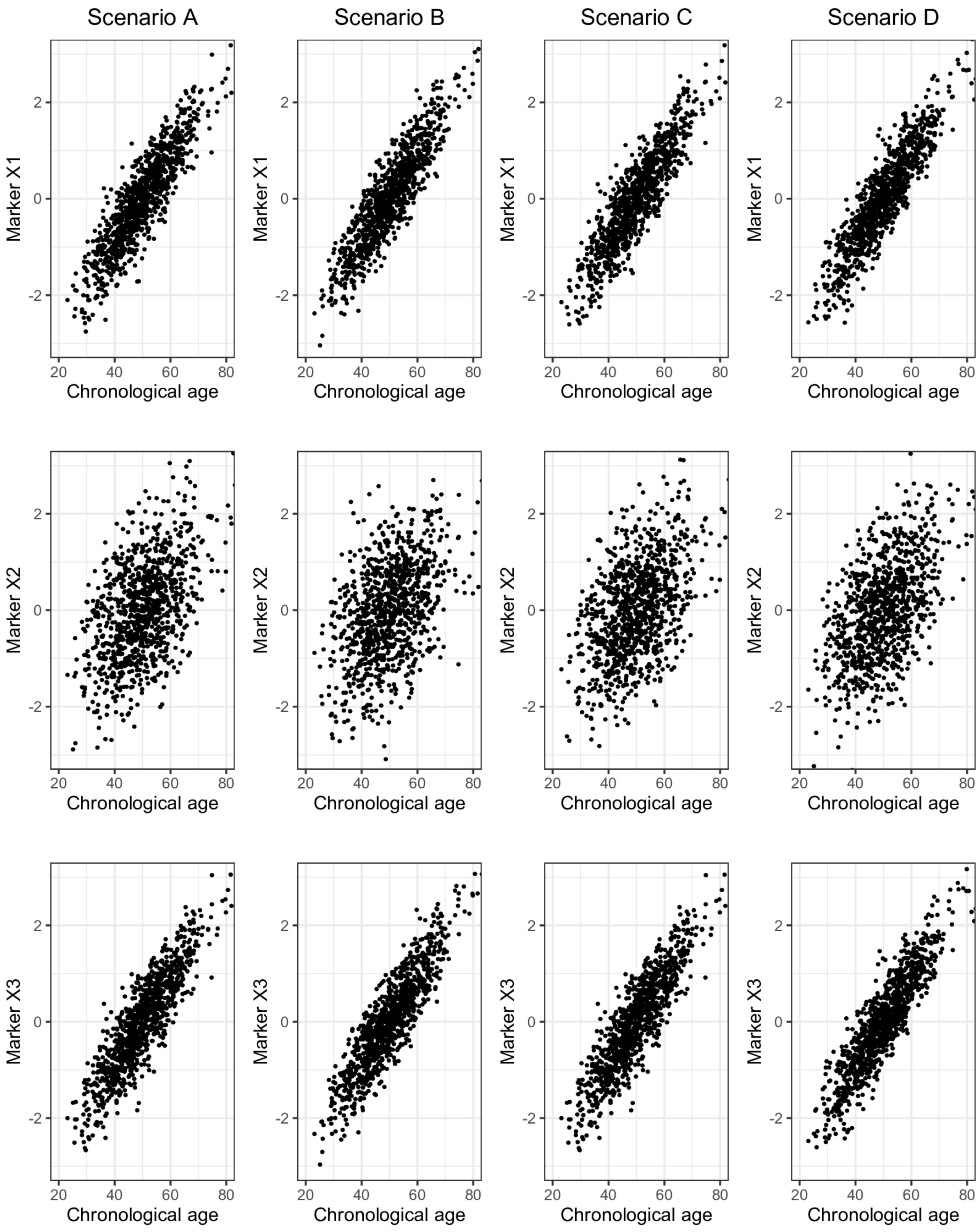

Supplementary Fig. 1. Scaled and centered markers X1, X2 and X3 plotted against chronological age for scenarios A (identical-association assumption holds), B (inverse association), C (no association) and D (identical-association assumption partially holds).
